# Characteristics of martial art injuries in a defined Canadian population: a descriptive epidemiological study

**DOI:** 10.1186/1471-2458-10-795

**Published:** 2010-12-30

**Authors:** Mark McPherson, William Pickett

**Affiliations:** 1 Department of Community Health and Epidemiology, Queen's University, Kingston, Canada; 2 Department of Emergency Medicine, Queen's University, Kingston, Canada

## Abstract

**Background:**

The martial arts have emerged as common activities in the Canadian population, yet few studies have investigated the occurrence of associated injuries on a population basis.

**Methods:**

We performed such an investigation and suggest potential opportunities for prevention. The data source was 14 years (1993 to 2006) of records from the Kingston sites of the Canadian Hospital Injury Reporting and Prevention Program (CHIRPP).

**Results:**

920 cases were identified. Incidence rates were initially estimated using census data as denominators. We then imputed annual injury rates per 10000 using a range of published estimates of martial arts participation available from a national survey. Rates of injury in males and females were 2300 and 1033 per 10000 (0.3% participation) and 575 and 258 per 10000 (1.2% participation). Injuries were most frequently reported in karate (33%) and taekwondo (14%). The most common mechanisms of injury were falls, throws and jumps (33%). Fractures (20%) were the most frequently reported type of injury and the lower limb was the most common site of injury (41%).

**Conclusions:**

Results provide a foundation for potential interventions with a focus on falls, the use of weapons, participation in tournaments, as well as head and neck trauma.

## Background

*Martial Arts *is a general term that describes the art of combat and self-defense. These arts involve the use of different body parts and various weapons [1]. A large variety of martial arts exist, each with a distinct history, philosophy and set of techniques. In the Kingston area of Canada, several of these styles are taught and practiced by participants of all ages. Benefits of participation include discipline, self-defense and engagement in physical activity. Injury risks associated with participation, however, are not well defined.

The popularity of martial arts has flourished over the past fifteen years [2]. Consequently, the number of injuries attributed to martial arts has also escalated [3]. Sport injuries result from acute trauma or repetitive stress associated with participation in athletic activities and can range from acute trauma to long-term disabilities [4]. Injuries in martial arts are common [5], especially amongst young adults [6], and thus may pose a potential public health concern.

Development of potential prevention strategies for martial arts injuries requires characterization of injured participants as well as assessments of the types of injuries sustained. In 2005, Zetaruk *et al *classified each injury by style of martial art studied, and injuries were most common in Taekwondo and Aikido [2]. Across styles, commonly reported injury types include fractures, hematomas and contusions [1,5,6], with injuries of the lower limb being most common [1,2]. Injury profiles commonly differ between styles; Yard *et al*. determined that participants in Judo sustained higher proportions of neck injuries and shoulder/upper arm injuries [7]. Few other studies have examined these issues at the population level, and no such studies have been conducted in Canada.

Emergency department surveillance can provide a basis from which to profile injuries at the population level. We had the opportunity to use a population-based injury surveillance system to describe the incidence of martial arts injuries in a defined Canadian population, and also describe key patterns associated with these injuries. Our hope was to provide an evidence base for prevention efforts.

## Methods

The Canadian Hospitals Injury Reporting and Prevention Program (CHIRPP) is an ongoing hospital-based surveillance program that registers injuries and poisoning cases from sixteen Canadian hospital emergency departments [8]. Upon arrival to the emergency department, the subject or caregiver fills in a one page report describing the injury or poisoning event. In severe cases, when the subject is unable to complete the form, attending research staff fill in the report after patient interviews [8]. Questions on the form describe the circumstances surrounding the injury, including the factors causing or contributing to the injury and the time and place of the injury event. Medical diagnostic information is coded by a nurse coordinator. This information includes the nature and location of the injury as well as the treatment provided [8]. Quality control to ensure the capture and coding of all injury and poisoning events is continuous. To ensure data capture, the emergency room registration staff ask each case if they have been injured or poisoned, and the subject or caregiver is immediately provided with a CHIRPP form in the affirmative. All data forms are compiled confidentially by trained data entry clerks from the Public Health Agency of Canada [9]. The data program undergoes regular logic checks to detect unlikely information combinations to ensure data quality [8].

Kingston, Ontario has two emergency departments located at the Hotel Dieu and Kingston General Hospitals. Both of these urgent care facilities are included in the Kingston CHIRPP registry, which began data collection in 1993. The Kingston CHIRPP catchment area also supports the surrounding area including Frontenac, Lennox and Addington counties [10]. Based upon Census data projections, the total population served by the Kingston hospitals that participate in CHIRPP is approximately 188 000 [10]. Because it incorporates the only two emergency departments in the area, Kingston CHIRPP can be considered representative of all injuries and poisoning events requiring urgent care in the population.

Patients presenting to the Kingston General Hospital or the Hotel Dieu (Kingston) emergency departments between Jan 1 1993 and Dec 31 2006 were included. Repeat visits were included and treated as independent injury events. Inclusion criteria were: (1) mechanism codes on text descriptions that contained one of the following terms: martial arts, karate, jujitsu, taekwondo, judo, kickboxing, tai chi, sparring or (2) coded with the CHIRPP *factor code *for Martial Art injuries. Despite differences in training and philosophy between the styles, namely tai chi and aikido, injuries in either style were included. This study obtained ethics approval by the Health Science Research Ethics Board of Queen's University.

### Analysis

Age standardized incidence rates per 10000 inhabitants were calculated by gender for each year of the study. Next, age and gender-specific incidence rates were calculated with 95% confidence intervals based on the normal approximation to the binomial [11]. Numerators were obtained from the hospital-based CHIRPP surveillance data, with denominators from the 2006 Canada Census of the Population [10]. We refer to these as "crude" rates in our analysis. To our knowledge, martial art participation rates are unavailable for the Kingston area and consequently we imputed injury rates using denominators based upon Canadian participation estimates cited in the Canadian General Social Survey [12] in both the active (1.2% participation) and the general (0.3% participation) population.

Descriptive statistics were used to profile key characteristics of the injuries sustained: demographics, timing, severity, cause and nature of the injury. Severity was determined by disposition from the emergency room, with severe injuries requiring hospital admittance or further follow-up care, as suggested by the attending physician. Details regarding the style of martial art and the mechanism of injury were obtained through written descriptions of the injury event. In order to ensure coding accuracy for data elements abstracted from the narrative records, two coders coded a random selection of 100 cases, and Kappa coefficients were calculated to ensure reliability in coding between raters. These coefficients ranged from good (k = 0.68) to perfect (k = 1.00) for the five key variables. Data management and analyses were performed in Microsoft Excel (Microsoft Corp, 2003) and SPSS (SPSS Corp, 2006). Proportions were compared using chi-square tests; an alpha value of 0.05 was used for all hypothesis testing. Key patterns were also described qualitatively based upon reviews of narrative text descriptions. Illustrative vignettes were used to highlight key patterns of injury.

## Results

### Rates of Injury

A total of 920 injury records met the inclusion criteria. The average annual number of injuries was 65.7 (*range*: 26 - 108) per year. The "crude" age-standardized incidence rate between 1993 and 2006 was 6.0 per 10000 people, per year (95% CI: 5.6 - 6.3) in the entire population of Kingston. Imputed rates based upon two estimates of participation in Kingston were considerably higher. Analogous crude and imputed injury rates for males and females are also presented for four age groups in Table 1.

**Table 1:** Crude and imputed annual rates of martial arts injury in Kingston, 1993-2006.

	Age-standardized rate per 10,000 (95% CI)											
Rate	Males						Females					
	Crude		0.3% Participation		1.2% participation		Crude		0.3% Participation		1.2% participation	
Overall	6.9	(6.4-7.5)	2300	(2133-2500)	575	(533-625)	3.1	(2.8-3.5)	1033	(933-1167)	258	(233-292)
By Age Group												
<19	4.8	(5.1-5.5)	1600	(1367-1833)	400	(425-459)	4.4	(4.1-5.5)	1467	(1367-1833)	367	(342-458)
20 - 44	13.7	(12.3-15.0)	4567	(4100-5000)	1141	(1025-1250)	4.7	(4.0-5.4)	1567	(1333-1800)	392	(333-450)
45 - 64	1.5	(1.1-1.9)	500	(36 - 633)	125	(92-158)	1.0	(0.7-1.3)	333	(233-433)	83	(58-108)
65+	*						*					

*Crude rates represent rates per 10,000 population. Imputed rates based upon 0.3 to 1.2% participation in martial arts *^┼ ^*are also presented. All rates are age-standardized*.

* not estimated due to small number of events

^┼ ^Rates of participation estimated from the General Social Survey [12]

**Figure 1:**
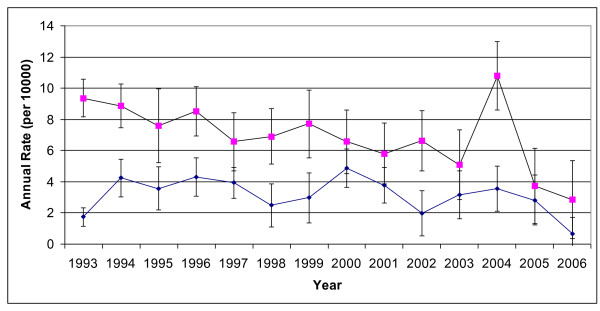
**Age Standardized incidence rates of martial art injuries in Kingston and area, 1993 - 2006, based on the crude (non-imputed) data**. Square data points represent incident rates in males, and circular shaped points represent the rates in females. 95% confidence intervals are indicated. *Denominators extrapolated from Census data, and the median year (1999) was used as the standard population.

Age-standardized incidence rates for both sexes are shown in Figure 1. Males showed consistently higher incidence rates than women, and showed a peak incidence in 2004. In 2006, female incidence rates were seen to drop by 2.0 per 10000 people.

### Injury Characteristics

The number, percentage and prevalence ratios of the demographics and timing of the injuries are profiled in Table 2. Nearly three quarters of the injuries (684/920) occurred on a weekday, and there was very little difference in the number of reported injuries with respect to the time of year. Karate injuries were the most prominent (32.5% [299/920]), while injuries due to tai chi were least reported (0.5% [5/920]).

**Table 2:** Demographics and timing of martial arts injuries in Kingston and Area, 1993 - 2006

	N	%	**PR**^**†**^**(95% CI)**
**Gender**			

Male	632	69	1.00
Female	288	31	0.44 (0.41-0.50)

**Age**			

<19	267	29	1.00
20 - 44	568	62	2.13(1.9-2.3)
45 - 64	80	9	0.31(0.24-0.37)
65+	5	1	NA^‡^

**Day of Week**			

Weekday	684	74	1.00
Weekend	236	26	0.35 (0.31-0.39)

**Style**			

Karate	299	33	1.00
Taekwondo	129	14	0.42(0.37-0.50)
Judo	99	11	0.36(0.28-0.40)
Kickboxing	60	7	0.21(0.16-0.25)
Jujitsu	46	5	0.15(0.12-0.20)
Aikido	16	2	0.06 (0.03-0.09)
Kung Fu	15	2	0.05 (0.03 - 0.08)
Tai Chi	5	1	NA^‡^
Unknown	251	27	0.84 (0.75-0.95)

**Location**			

Training Facility	461	50	1.00
Tournament/Demonstration	186	20	0.40 (0.35-0.46)
Residential	79	9	0.18 (0.14-0.21)
School	16	2	0.03 (0.02-0.06)
Unknown	178	19	0.38 (0.38-0.44)

**Severity**			

Severe*	236	26	1.00
Mild	684	74	0.35 (0.31-0.39)

* Admitted or required follow-up.

‡ Not estimated due to small number of events

^† ^PR, Prevalence Ratio

Of the injuries sustained by male subjects, 55.1% (507/920) were reported as being inflicted by another. More female subjects sustained self-inflicted injuries (41.0% [118/288]), such as fractured limbs from a fall or foot injuries from dropped weapons. The highest number of self-inflicted injuries was reported in the youngest age group; <19 years (53.1% [110/207]). Of the injuries sustained by those older than 65, 60% (3/5) were inflicted by another participant.

The most common mechanism of injury was a fall, throw or jump (32.7% [301/920]) (Table 3). Injuries caused by weapons were most serious. The most serious injuries were recorded in kung fu (40% [6/15]); aikido and jujitsu had the lowest percentages.

**Table 3:** The gender and severity of injuries by style of martial arts, cause/mechanism and nature of injury sustained

	**n**^**† **^**(%)**	% Male	% Serious*
**Style**			

Karate	299 (33)	65	25
Taekwondo	129 (14)	67	28
Judo	99 (12)	73	27
Kickboxing	60 (7)	65	25
Jujitsu	46 (5)	83	20
Aikido	16 (2)	63	13
Kung Fu	15 (2)	47	33
Tai Chi	5 (1)	60	40
Other	251 (27)	72	26

**Cause/Mechanism**			

Fall/Throw/Jump	301 (33)	65	27
Foot Strike/Kick	294 (32)	72	19
Hand Strike/Punch	77 (8)	74	34
Weapon	50 (5)	72	84
Block	47 (0)	64	19

**Nature of Injury**			

***Fracture***	189 (21)	65	67
Upper Limb	95 (10)	64	84
Lower Limb	66 (7)	67	15
Head & Neck	17 (2)	59	12
Trunk	10 (1)	60	0
Other	1 (0)	100	0
***Sprain/Strain***	112 (12)	71	14
Upper Limb	26 (3)	73	12
Lower Limb	70 (8)	67	19
Head & Neck	6 (1)	100	0
Trunk	9 (1)	67	0
Other	1 (0)	100	0
***Open Wound***	45 (5)	67	64
Upper Limb	5 (1)	40	40
Lower Limb	6 (1)	67	50
Head & Neck	32 (4)	69	66
Trunk	0	N/A	N/A
Other	2 (0)	100	0

* Admitted or required follow-up.

^† ^Numbers may not total 920 due to missing or incomplete data

The use of protective gear was reported in 294 (32%) subjects. The severity of injuries in these subjects was very similar to those who did not report the use of any protection (p = 0.77). 24.8% (73/294) of people with protection sustained a severe injury, and 25.9% (161/621) of those who did not claim any protection use had a severe injury. The odds of sustaining a severe injury in cases who used protective gear did not differ from those with no reported protection (OR = 1.06, 95% CI:0.81-1.45).

The severity of common types of injuries in the five styles with the highest incidence rates are shown by gender in Table 4. Fractures were the most common injury reported, nearly half of which were reported in karate (72/189). These injuries were most serious in karate; 74% (53/72) were admitted or required follow-up care.

**Table 4:** The gender and severity of injuries reported in 5 types of injury and martial art styles

	**n**^**† **^**(%)**	% Male	% Serious*
**Fracture**	**189 (21)**	**65**	**67**

Karate	72 (8)	68	74
Taekwondo	27 (3)	74	52
Judo	18 (2)	56	50
Kickboxing	14 (2)	71	50
Jujitsu	6 (1)	88	67

**Sprain/Strain**	**112 (12)**	**71**	**14**

Karate	42 (5)	62	7
Taekwondo	13 (1)	62	23
Judo	12 (1)	67	0
Kickboxing	2 (0)	50	50
Jujitsu	7 (1)	86	0

**Dislocation**	**46 (5)**	**58**	**20**

Karate	16 (2)	56	25
Taekwondo	9 (1)	88	11
Judo	5 (1)	40	60
Kickboxing	3 (0)	67	33
Jujitsu	1 (0)	100	0

**Open Wound**	**45 (5)**	**67**	**64**

Karate	11 (1)	82	82
Taekwondo	8 (1)	75	50
Judo	6 (1)	83	83
Kickboxing	4 (0)	75	25
Jujitsu	2 (0)	100	50

**Head Injury**	**28 (3)**	**86**	**35.7**

Karate	10 (1)	80	40
Taekwondo	7 (1)	86	43
Judo	3 (0)	67	67
Kickboxing	3 (0)	100	33
Jujitsu	1 (0)	100	0

* Admitted or required follow-up

^† ^Numbers may not total 920 due to missing or incomplete data

Table 5 depicts the mechanisms of injury associated with each style. Falls, throws and jumps were the most frequent cause of injury in most styles other than karate, kickboxing and taekwondo, in which kicks and foot strikes were the sources of the greatest morbidity. Blocks and weapons were the cause of the least number of injuries in each style.

**Table 5:** Number and Percentage of injuries [n(%]) caused by five mechanisms in each martial art style studied (Row Percent)

Style	Fall/Throw/Jump		Kick/Foot Strike		Punch/Hand Strike		Block		Weapon	
	**n**	**%**	**n**	**%**	**n**	**%**	**n**	**%**	**n**	**%**

Karate (n = 299)	77	26	115	38	29	10	28	9	15	5
Taekwondo (n = 129)	30	23	68	53	8	6	4	3	11	9
Judo (n = 99)	65	66	3	3	1	1	0	0	1	1
Kickboxing (n = 60)	6	3	30	50	10	16	2	3	1	2
Jujitsu (n = 46)	24	52	6	13	2	4	0	0	1	2
Aikido (n = 16)	9	56	0	0	0	0	0	0	3	19
Kung Fu (n = 15)	9	60	3	20	1	7	0	0	2	13
Tai Chi (n = 5)	3	60	0	0	1	20	0	0	0	0
Unknown (n = 251)	78	31	71	28	25	10	13	5	19	8

*Percentages do not add up to 100% because of missing or incomplete data

### Illustrative Vignettes

Recurrent patterns of injury identified during the present study are further described through the use of short hypothetical vignettes. These describe typical patterns of injury as inferred from the quantitative results, and characterize why these patterns are important to the various martial arts.

#### Pattern 1 -Falls and Throws

*During a Taekwondo practice session, a 14- year-old boy was practicing knee kicks with a partner when he lost his footing and fell onto the padded ground. The next day he was brought to the emergency room with a twisted ankle and was treated without requiring any further follow-up*.

Falls were commonly reported as injury mechanisms in subjects practicing at a training club. Twisted ankles and lower limb fractures were commonly associated with these falls. This event was typical because: a) Falls frequently occurred while working with a partner; b) Most falls did not require hospital admittance or any further follow-up; c) Falls were often a result of a loss of balance after a kick.

#### Pattern 2 - Weapons

*A 40-year-old man was practicing judo at his cottage. He was training with a pair of bokken [Japanese wooden swords], and he lost control of them and one flew into the air and hit his leg. He reported to the hospital later that day and was admitted because of an open wound to his knee as well as his foot*.

Weapon training can be incorporated into most styles of martial arts. The most commonly reported weapons inducing injury include the bokken (Japanese wooden sword of variable lengths), and the bō (3 cm thick wooden stick). This injury was typical because: a) Most weapon injuries were self inflicted; b) Injuries from weapons were commonly serious, requiring follow-up or hospital admission; c) Losing control of a weapon often resulted in multiple injuries.

#### Pattern 3 - Demonstrations and Tournaments

*Two age-matched adults were sparring in a karate demonstration. One participant was more experienced and had a black belt, while the other had less experience in the sport. The black belt punched her partner forcefully, who was unable to properly block the attack. The partner sustained a hand fracture, and required follow-up orthopaedic care*.

Martial art demonstrations provide a chance for participants to showcase their skills to family and friends. Demonstrations often involve participants with different ranks and levels of experience practicing together, which yield injuries when one of the participants is being more forceful than the other. This vignette was typical because: a) The participant with less experience sustained the injury; b) Inadequate blocking of a punch caused a fracture of the upper limb; c) No protection was reported while sparring; d) Fractures were the most commonly reported injury in karate.

#### Pattern 4 - Head and Neck Injuries

*A 14- year-old girl and her partner were practicing jujitsu by throwing each other onto the padded ground of a school gym. During one throw, the participant twisted her leg while falling, causing her to land on her head*.

Head and neck injuries were a major source of hospital admission and the requirement for professional follow-up. They were commonly a consequence of an impact with the ground. They resulted in open wounds, pulled neck muscles and concussions. This incident was typical because: a) A throw by another participant caused the other to hit the ground; b) Padding on the ground did not prevent the injury; c) Throws and falls were the most commonly reported injuries in jujitsu.

## Discussion

The participation-based rates indicate that a substantial number of martial art participants require emergency room care due to injury. These rates are comparable to previous smaller cohort studies of martial art practitioners [13,14], despite difference in study design. Injuries were more commonly reported for males than females, which is consistent with other surveillance studies [6,7]. The rates are population based, and provide useful public health information by identifying the number of emergency room visits attributable to martial art injury in the Kingston area.

By style, karate appears to be associated with the most injuries, although this observation does not account for style-specific rates of participation which remain unavailable for our population. Similarly, the use of protection was involved in many injury events. It is plausible that this protection was associated with more inherently dangerous maneuvers specific to martial arts styles. It is also possible that an increased perception of safety occurs among participants who use protective equipment, causing them to engage in higher risk activities. Similar findings have been observed elsewhere [15,16].

Strengths of this study included its novelty for Canada, the population-based nature of the analysis, our use of objective diagnostic data, and the conduct of analyses that were age, sex and style-specific. Limitations included our reliance on emergency department records, as individuals reporting to the emergency department may differ systematically from other injured participants. Further, the CHIRPP database was not designed specifically for the study of martial art injuries, and hence some event descriptions have limited detail about the circumstances leading to injury. For example, the style of martial art was not included for some injury events, and terminology was used inappropriately in other event descriptions. In addition, prevalence estimates were likely underestimated, as injured participants who did not report to the emergency department could not be included in the analyses.

One standard approach to the conceptualization of injury prevention strategies is the use of the three E's for injury prevention: (1) Education, (2) Engineering and (3) Enforcement [17]. Education uses behavioral interventions in order to prevent the events that may lead to an injury [18]. The severity, location and mechanisms of injury varied by each style of martial art, so it is clear that education must be tailored to each style. Over half of the total injuries were inflicted by another participant, thus prevention should focus on promoting safe practice between participants to reduce these injuries. Active protection, which requires the practitioners themselves to participate in preventive efforts, might play an important role in reducing injuries related to martial arts. This study was unable to fully characterize the utility of protective devices such as helmets and mouth guards in preventing injuries, though other studies have suggested that they may be useful in reducing injuries in martial arts [16,19].Contact sports have been shown to result in increased injury rates [13], so minimizing contact may be an effective prevention strategy to reduce these injuries.

Engineering requires environmental changes to reduce the total number and severity of injuries through passive protection [18]. Passive protection strategies are those that do not require the actions of the individual or participant in order for protection to occur [18]. In this study falls, throws and jumps were the leading mechanism of injury in jujitsu. An appropriate means of passive protection could be the implementation of floor padding in the training site. Additional research investigating such preventive instruments in each style is essential to determine their efficacy.

Enforcement requires the efforts of instructors and referees in assuring safe practices [18]. Over 70% of injuries were reported at a training site or at a competition, so instructors and referees do have the ability to regulate activities that may lead to morbidity. Safe practice guidelines for sports and physical activity have been developed for schools [20], and these may prove a useful resource for instructors and referees to minimize injuries in practitioners of all ages.

## Conclusion

This study was able to describe the magnitude and characteristics of martial art injuries in Kingston and area using emergency department injury records. Martial art injuries were found to be an important source of morbidity within the population, and the characteristics of these injuries provide a foundation for potential safety interventions to reduce the number of these injuries in the future.

## Competing interests

The authors declare that they have no competing interests.

## Authors' contributions

MM and WP conceived the study and performed all writing, statistical analysis and interpretation. Both authors read and approved the manuscript.

## Pre-publication history

The pre-publication history for this paper can be accessed here:

http://www.biomedcentral.com/1471-2458/10/795/prepub
